# ELAVL1‐dependent SOAT2 exacerbated the pancreatitis‐like cellular injury of AR42J cells induced by hyperstimulation with caerulein

**DOI:** 10.1002/kjm2.12911

**Published:** 2024-11-26

**Authors:** Yu‐Jing Sun, Hua‐Ying Chen, Xiao‐Qin Lai

**Affiliations:** ^1^ Emergency Department Zhongshan Hospital of Xiamen University Xiamen Fujian China

**Keywords:** ELAVL1, NRF2/HO‐1 pathway, pancreatitis, SOAT2

## Abstract

Pancreatitis is a severe inflammatory condition characterized by damage to the pancreas. Sterol o‐acyltransferase 2 (SOAT2) has been reported to aggravate acute pancreatitis, however, the underlying mechanism remains to be elucidated. Rat pancreatic exocrine cells (AR42J) were treated with caerulein to induce pancreatitis‐like cellular injury. Cell viability was determined using a cell counting kit‐8 (CCK‐8) assay, while cell proliferation was analyzed through a 5‐Ethynyl‐2′‐deoxyuridine assay. Cell apoptosis was measured using flow cytometry, and enzyme‐linked immunosorbent assays were performed to detect levels of pro‐inflammatory cytokines IL‐6 and TNF‐α. Additionally, Fe^2+^ levels were analyzed using a colorimetric assay kit, reactive oxygen species (ROS) levels were assessed with a Cellular ROS Assay kit, and lipid peroxidation was measured using a malondialdehyde assay kit. Glutathione levels were analyzed with a detection assay. Protein and mRNA expression were evaluated through western blotting and quantitative real‐time polymerase chain reaction, respectively. Furthermore, an RNA immunoprecipitation assay was conducted to investigate the association between ELAV‐like RNA binding protein 1 (ELAVL1) and SOAT2. Actinomycin D assay was performed to explore the effect of ELAVL1 depletion on the transcript stability of SOAT2 mRNA. SOAT2 and ELAVL1 expression were upregulated in caerulein‐exposed AR42J cells. Caerulein treatment induced pancreatitis‐like cellular apoptosis, inflammatory response, ferroptosis, and cell proliferation inhibition. Silencing of SOAT2 protected against caerulein‐induced AR42J cell injury. Moreover, ELAVL1 stabilized SOAT2 mRNA expression in AR42J cells. SOAT2 overexpression attenuated the effects induced by ELAVL1 silencing in caerulein‐exposed AR42J cells. Additionally, ELAVL1 knockdown activated the NRF2/HO‐1 pathway by downregulating SOAT2 expression in caerulein‐exposed AR42J cells. SOAT2 silencing protected AR42J cells from caerulein‐induced injury by inactivating the NRF2 pathway. In conclusion, ELAVL1‐dependent SOAT2 exacerbated pancreatic exocrine cell injury by inactivating the NRF2/HO‐1 pathway in pancreatitis. These findings provide new insights into the molecular mechanisms underlying pancreatitis and offer potential therapeutic targets for the treatment of this condition.

## 1 INTRODUCTION

Acute pancreatitis is a severe inflammatory condition of the pancreas that can be caused by various factors, including alcohol abuse, gallstones, and infections.^1^ This disorder can be sudden and severe, causing severe pain in the abdomen and other symptoms such as nausea, vomiting, and diarrhea.^2^, ^3^ Acute pancreatitis can lead to various complications, including necrosis, pancreatic infection, bleeding, and fluid accumulation that can require surgical intervention.^4^, ^5^ Acute pancreatitis may increase the risk of pancreatic ductal adenocarcinoma.^6^, ^7^ Despite significant advancements made in understanding the pathology and physiology of acute pancreatitis, there are still significant gaps in our understanding of effective treatment strategies for acute pancreatitis.^8^ To address this gap, it is crucial to explore the deeper mechanisms behind acute pancreatitis to develop more targeted treatments and preventative measures.

Sterol o‐acyltransferase 2 (SOAT2) plays a crucial role in the metabolism of cholesterol and other sterols in the body.^9^ SOAT2 is responsible for catalyzing the esterification of cholesterol, converting it into cholesteryl esters. This process is essential for the storage and transportation of cholesterol within cells. SOAT2 has been implicated in various metabolic disorders, such as atherosclerosis and non‐alcoholic fatty liver disease.^10^ A recent paper revealed that SOAT2 inhibitor alleviated acute pancreatitis,^11^ however, the molecular mechanism underlying how SOAT2 regulates acute pancreatitis remains unclear. Understanding the mechanism of SOAT2 in acute pancreatitis can provide valuable insights into potential targets for intervention and the development of novel therapeutic approaches for managing this metabolic disorder.

ELAV‐like RNA binding protein 1 (ELAVL1), also named HUR, is an RNA‐binding protein that belongs to the ELAV family of RNA‐binding proteins and plays an essential role in the regulation of gene expression in cells.^12^ ELAVL1 can bind to specific sequences in RNA through its RNA‐binding domain and thus regulates the expression of target genes by influencing the translation or degradation of RNA.^13^ ELAVL1 is involved in the regulation of various biological processes in cells, including cell cycle progression, apoptosis, and cranial neural crest specification.^14^, ^15^, ^16^ It has been shown to play a role in cancer development, where it can promote cell proliferation and tumorigenesis.^17^ It is also implicated in the development of autoimmune diseases and other diseases with an inflammatory component.^18^, ^19^ In particular, ELAVL1 may modulate the expression of certain genes related to inflammation in response to pancreatic injury,^20^ indicating its involvement in pancreatic inflammation.

NRF2 is a transcription factor that promotes the expression of antioxidant and detoxification genes in response to oxidative stress, thereby reducing the damage caused by reactive oxygen species (ROS).^21^, ^22^, ^23^ HO‐1 is an enzyme that catalyzes the degradation of heme into biliverdin, iron, and carbon monoxide.^24^ The NRF2/HO‐1 pathway has been shown to regulate pancreatic injury.^25^ The ENCORI online database identified ELAVL1 as an RNA‐binding protein of SOAT2. Therefore, we constructed the ELAVL1/SOAT2/NRF2/HO‐1 axis to elucidate the mechanism underlying SOAT2's regulation in the progression of acute pancreatitis, with the goal of identifying potential therapeutic targets for this disorder.

## 2 MATERIALS AND METHODS

### 2.1 In vitro cell model

Rat pancreatic exocrine cells (AR42J, EK‐Bioscience, Shanghai, China) were maintained in F12K medium (EK‐Bioscience) with 20% fetal bovine serum (EK‐Bioscience) and 1% penicillin/streptomycin (Cytiva, Shanghai, China) at 37°C with 5% CO_2_. Caerulein (MedChemExpress, Princeton, NJ, USA) was prepared with phosphate buffered solution (PBS) and then diluted to the desired working concentration (5, 10 and 15 nmol/L) with the final medium. The prepared caerulein was added into the cell culture wells, while the control group was added with an equal volume of PBS. Cells were cultured in a 37°C, 5% CO_2_ cell culture incubator for 24 h to establish an in vitro model of pancreatitis.^26^

### 2.2 Cell transfection

Small interfering RNAs of SOAT2 (si‐SOAT2) and ELAVL1 (si‐ELAVL1), SOAT2 overexpression plasmid (constructed using pcDNA 3.1 vector), and respective controls (si‐NC and vector) were provided by Songon Biotech (Shanghai, China). Cells were harvested when they had reached approximately 90% confluence and were seeded into 12‐well plates. Transfection was performed 15 h after plating, with fresh F12K medium being replaced 1 h and a half before transfection. The cells were allowed to stabilize at 37°C in the incubator. Lipofectamine 3000 (Thermo Fisher, Waltham, MA, USA) was diluted in opti‐MEM (Sigma, St. Louis, MO, USA), and siRNA or overexpression plasmids were also dissolved in opti‐MEM medium. These two solutions were mixed, and after removing the cells from the incubator, the mixture was gently added to the culture medium and returned to the incubator. For experiments requiring the construction of a cell model with caerulein (MedChemExpress), the transfected cells were washed and the prepared caerulein was added to each well.

### 2.3 Cell treatment

To analyze whether the regulation of SOAT2 in caerulein‐induced cell injury involved NRF2, AR42J cells were treated with a specific NRF2 inhibitor ML385 (5 μM, MedChemExpress) before modeling and after transfection. Then, the cells were harvested for further functional assays.

### 2.4 Cell viability analysis

The transfected AR42J cells were resuspended, and cell suspension was slowly loaded onto the sample wells of the cell counting plates. Based on the calculated cell concentration, 3000 cells were added to each well of the 96‐well plates, and the cells were gently dispersed by tapping after adding the culture medium. After culturing the cells for 48 h, the 96‐well plates were removed, and the original culture medium was discarded. CCK‐8 (Abcam, Cambridge, UK) diluted with serum‐free culture medium was added, and the cells were then further incubated for 2 h. The samples were analyzed using a microplate reader.

### 2.5 Cell proliferation analysis

AR42J cells were resuspended and seeded into 96‐well plates. After the cells were attached to the plates, experimental groups were established by adding plasmids for transfection and/or adding caerulein (MedChemExpress) for stimulation. Each well was then incubated with medium containing EdU (Ribobio, Guangzhou, China) for 2 h. Cell fixation solution was added to each well and incubated for 30 min. Glycine solution (Ribobio) was added to each well, and the plates were incubated on a shaker for 5 min. After PBS washing, 1× Apollo staining reagent (Ribobio) was added to each well, and the plates were incubated on a shaker for 30 min. The cells were then stained with DAPI (Sigma). Immediately after staining, fluorescence microscopy was used to detect the cells.

### 2.6 Analysis of apoptotic cells

After cell transfection and modeling were completed, cells were washed once with PBS solution, followed by the addition of an appropriate amount of pancreatic enzyme digestion solution (Solarbio, Beijing, China). Binding Buffer (Solarbio) was added to gently resuspend the cells, and then Annexin V‐FITC (Solarbio) was added for incubation. PI staining solution (Solarbio) was then added, and the mixture was gently swirled. The plates were placed in an ice bath for 5 min, and flow cytometry was performed within 30 min.

### 2.7 Enzyme‐linked immunosorbent assays

Before detection, the cell culture supernatant and the reagents in the kit were slowly thawed at room temperature. The operation was carried out according to the instructions of the Rat IL‐6 and TNF‐α Elisa Kit (#abs530003 and #abs530007, Absin Biotech, Shanghai, China). The 96‐well plates provided in the kits were taken out, and wash Buffer and assay buffer were added to each well in sequence. The cell culture supernatant to be tested was added to other wells. After incubating for 1 h, the diluted antibodies in assay buffer were evenly added to each reaction well and incubated for 2 h. Horseradish peroxidase (HRP) solution was added to each well for color development in a dark room. After adding the stop buffer, the OD value was measured using a microplate reader.

### 2.8 Fe^2+^ concentration detection

According to the guidebook of the commercial Fe^2+^ detection kit (#E‐BC‐K881‐M, Elabscience, Wuhan, China), cells were harvested after cell transfection and modeling. Reagent I was added and cells were lysed for 10 min. After adding the samples to detection wells, microplate reader was used to analyze Fe^2+^ levels.

### 2.9 Reactive oxygen species detection

Low‐speed centrifugation was employed to facilitate the settling of the lyophilized powder (Solarbio) at the bottom of the container. PBS solution was diluted to a working concentration of 1–10 μM and added to the cells for 1 h. The staining working solution (Solarbio) was aspirated, and the cells were washed once with culture medium, followed by further incubation at room temperature. The background fluorescence intensity of the loaded cells was determined. The fluorescence of control cells cultured in growth medium or sample buffer was checked. The above procedures were performed according to the guidebook of a ROS detection kit (Solarbio).

### 2.10 Malondialdehyde content detection

PBS was mixed with cells at 4°C, and the supernatant was collected after centrifugation for subsequent measurements. PBS (blank control) was added to centrifuge tubes, and standard curve was prepared with different concentrations of standard samples. Later, samples were added for measurement, followed by the addition of malondialdehyde (MDA) solution (Beyotime). The mixture was heated in a boiling water bath for 15 min, and the supernatant was transferred to 96‐well plates and analyzed using a microplate reader.

### 2.11 Glutathione detection

According to the instructions provided with the GSH Assay Kit (Abcam), we conducted a thorough analysis of GSH levels in AR42J cells using a microplate reader.

### 2.12 Western blotting assay

Cell culture dishes were placed on ice, and the cells were lysed using RIPA lysis buffer (Beyotime). After centrifugation, the supernatant was transferred to new EP tubes on ice, and the pellets were discarded. Gels were prepared using an SDS‐PAGE fast gel kit (Phygene, Fuzhou, China), loaded with samples, and subjected to electrophoresis. Transblotting was conducted in constant current mode, with a current setting of 290 mA for 90 min. PVDF membranes (Membrane Solutions, Shanghai, China) were incubated on a shaker in the blocking solution. The PVDF membranes were cut based on the approximate positions of the protein marker and target protein and then placed in the incubation solution including primary antibodies against SOAT2 (#PA5‐100373, 1:1000, Thermo Fisher), ELAVL1 (#PA5‐90917, 1:1000, Thermo Fisher), NRF2 (#PA5‐88084, 1:1000, Thermo Fisher), HO‐1 (#PA5‐77833, 1:1000, Thermo Fisher), GPX4 (#MA5‐32827, 1:10000, Thermo Fisher) and GAPDH (#PA1‐16777, 1:5000, Thermo Fisher). The PVDF membranes were placed in ECL working solution (Beyotime) and then exposed to the gel imaging system for visualization.

### 2.13 Quantitative real‐time polymerase chain reaction

To extract RNA, cells were removed from an incubator and the upper layer of culture medium was discarded. Trizol reagent (Beyotime) was added and allowed to stand for 5 min before transferring the liquid to EP tubes for RNA extraction. The extracted RNA was diluted and its concentration was determined. Based on the determined concentration, the volume of RNA solution for reverse transcription was calculated. The RNA solution was then used as a template for reverse transcription using the PrimeScript RT Enzyme Mix (Yeasen, Shanghai, China). After determining the cDNA concentration, SYBR Premix Ex TaqII (Yeasen) was used for quantitative analysis of SOAT2 and GAPDH expression. Finally, gene expression was analyzed using the 2^−∆∆Ct^ method. SOAT2 5′‐GATGGGCTGTGGCTCTTAGG‐3′ and 5′‐GTCGACGAGCATACCACTCC‐3′; GAPDH 5′‐GGTGAAGGTCGGTGTGAACG‐3′ and 5′‐CTCGCTCCTGGAAGATGGTG‐3′.

### 2.14 RNA immunoprecipitation assay

The ELAVL1‐RIP assay was performed using the antibody against ELAVL1 (#sc‐374,285, SantaCruz, SantaCruz, California, USA) or IgG (#ab172730, Abcam) according to the guidebook provided with the Magna RNA immunoprecipitation kit (Millipore). AR42J cells were harvested and subjected to lysis using RIPA lysis buffer (Beyotime). Then, the cell lysates were incubated with magnetic beads conjugated with antibodies for 24 h. SOAT2 mRNA expression was analyzed by quantitative real‐time polymerase chain reaction (qRT‐PCR).

### 2.15 Transcript half‐life analysis of SOAT2

AR42J cells with approximately 90% confluence were transfected with ELAVL1 siRNA and si‐NC using Lipofectamine 3000 (Thermo Fisher). After 24 h, Actinomycin D (Abcam) was diluted and added to each well for 3, 6, and 9 h. The cells were harvested for SOAT2 mRNA analysis by qRT‐PCR.

### 2.16 Statistical analysis

GraphPad Prism was used for statistical analysis, and results were shown as mean ± standard deviation (SD). Experimental comparisons were conducted using two‐tailed Student's *t*‐tests, or one (two)‐way analysis of variance (ANOVA). *p* < 0.05 was indicative of statistical significance.

## 3 RESULTS

### 3.1 Caerulein treatment induced pancreatitis‐like cellular apoptosis, inflammatory response, and ferroptosis and cell proliferation inhibition

**FIGURE 1:**
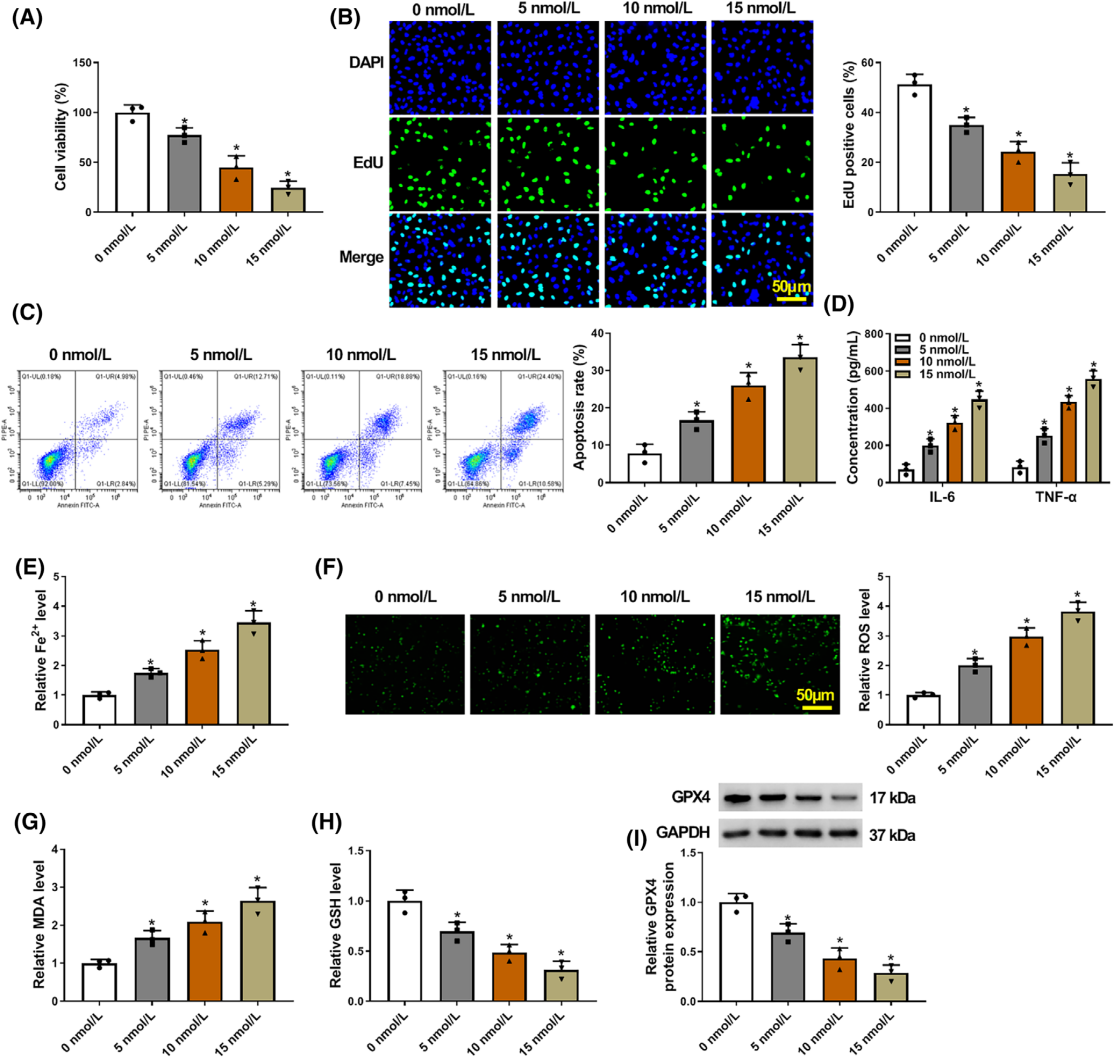
Caerulein treatment induced pancreatitis‐like cellular apoptosis, inflammatory response, and ferroptosis. AR42J cells were treated with PBS or caerulein (5, 10, and 15 nmol/L). (A) Cell viability was assessed by the CCK‐8 assay. (B) Cell proliferation was analyzed by the EdU assay. (C) Cell apoptosis was assessed by flow cytometry. (D) ELISAs were performed to detect IL‐6 and TNF‐α levels. (E) Fe^2+^ colorimetric assay kit was used for Fe^2+^ level analysis. (F) ROS level was detected using a Cellular ROS Assay kit. (G) MDA level was analyzed using a lipid peroxidation MDA assay kit. (H) Glutathione detection assay was performed to analyze GSH level. (I) GPX4 protein expression was detected by western blotting assay. *p < 0.05.

AR42J cells were stimulated using caerulein (5, 10, and 15 nmol/L) to mimic pancreatitis‐like cellular injury. The results showed that caerulein treatment inhibited AR42J cell viability and cell proliferation and promoted cell apoptosis in a dose‐dependent manner (Figure 1A–C). Comparatively, the levels of IL‐6, TNF‐α, and Fe^2+^ were upregulated after caerulein treatment in a dose‐dependent manner (Figure 1D,E). Moreover, we discovered that caerulein‐induced AR42J cells showed increased ROS and MDA levels and decreased GSH and GPX4 levels (Figure 1F–I). About 10 nmol/L of caerulein was selected for subsequent assays because cell viability was reduced by 50% at this concentration. These data demonstrate that caerulein treatment can induce pancreatitis‐like cellular injury.

### 3.2 SOAT2 silencing protected against caerulein‐induced AR42J cell injury

**FIGURE 2:**
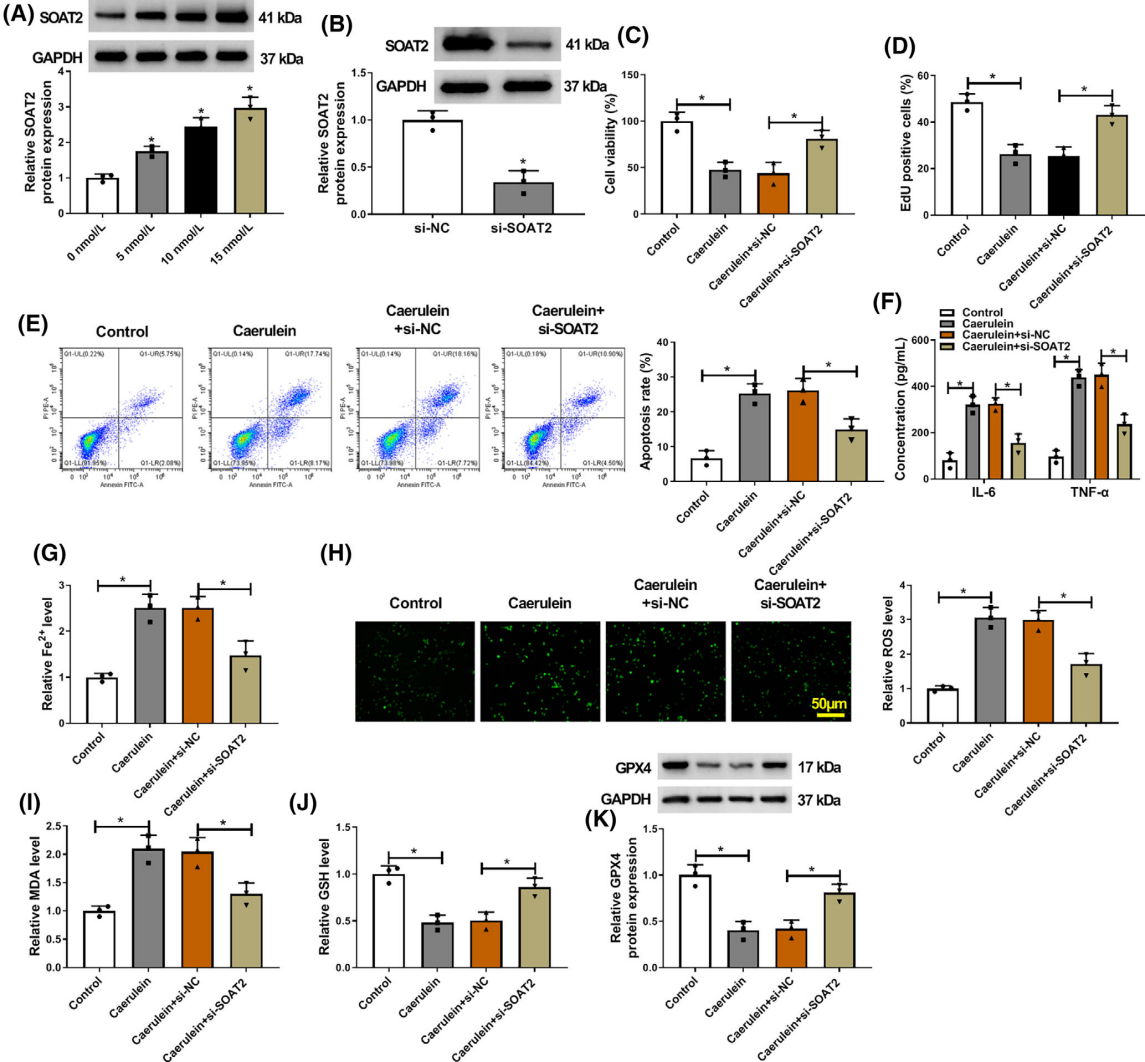
SOAT2 silencing protected against caerulein‐induced AR42J cell injury. (A) The effects of caerulein treatment (0, 5, 10, and 15 nmol/L) on SOAT2 protein expression were assessed by western blotting assay. (B) The efficiency of SOAT2 knockdown was determined by western blotting assay. (C–K) AR42J cells were divided into control group, caerulein group, caerulein+si‐NC group, and caerulein+si‐SOAT2 group. (C) Cell viability was assessed by the CCK‐8 assay. (D) Cell proliferation was analyzed by the EdU assay. (E) Cell apoptosis was assessed by flow cytometry. (F) ELISAs were performed to detect IL‐6 and TNF‐α levels. (G) Fe^2+^ colorimetric assay kit was used for Fe^2+^ level analysis. (H) ROS level was detected using a Cellular ROS Assay kit. (I) MDA level was analyzed using a lipid peroxidation MDA assay kit. (J) Glutathione detection assay was performed to analyze GSH level. (K) GPX4 protein expression was detected by western blotting assay. *p < 0.05.

The study then analyzed the effects of SOAT2 knockdown on caerulein‐induced AR42J cell injury by transfecting SOAT2 siRNA into caerulein‐exposed AR42J cells. The results first showed that caerulein treatment elevated SOAT2 protein expression in a dose‐dependent manner (Figure 2A). The high efficiency of SOAT2 knockdown was assessed by western blotting assay, and the result is shown in Figure 2B. Subsequently, the results showed that SOAT2 knockdown rescued caerulein‐induced inhibitory effects on cell viability and cell proliferation (Figure 2C,D). In addition, caerulein‐induced promoting effects on cell apoptosis and IL‐6, TNF‐α, and Fe^2+^ levels were counteracted after SOAT2 silencing (Figure 2E–G). Moreover, the increased ROS and MDA levels and decreased GSH and GPX4 levels induced by caerulein were also attenuated after transfection with siRNA of SOAT2 (Figure 2H–K). These results indicate that SOAT2 may aggravate caerulein‐induced AR42J cell injury.

### 3.3 ELAVL1 stabilized SOAT2 mRNA expression in AR42J cells

**FIGURE 3:**
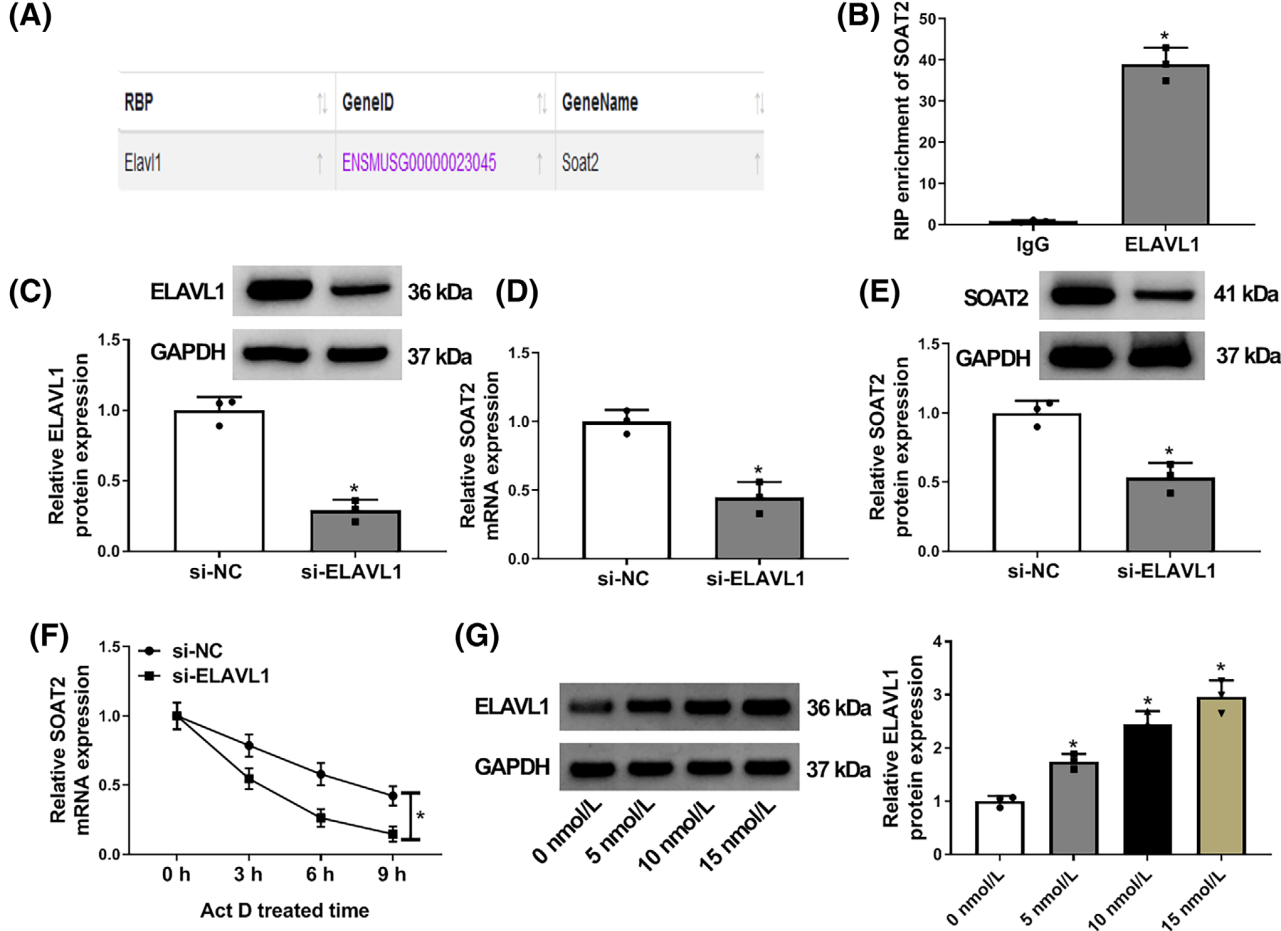
ELAVL1 stabilized SOAT2 mRNA expression in AR42J cells. The ENCORI online database was used to predict the RNA binding proteins of SOAT2. (B) The RIP assay was conducted to identify the association between ELAVL1 and SOAT2 in AR42J cells. (C) The efficiency of ELAVL1 knockdown was analyzed by western blotting assay in AR42J cells. (D and E) The effects of ELAVL1 silencing on SOAT2 expression were determined by quantitative real‐time polymerase chain reaction and western blotting assay. (F) Actinomycin D assay was performed to determine the effect of ELAVL1 depletion on the transcript half‐life of SOAT2 mRNA. (G) The effects of caerulein treatment (0, 5, 10, and 15 nmol/L) on ELAVL1 protein expression were assessed by western blotting assay. *p < 0.05.

The study continued to analyze the upstream regulator of SOAT2 through the ENCORI online database. As shown in Figure 3A, ELAVL1 was identified as an RNA‐binding protein of SOAT2. Subsequently, the RIP assay showed that SOAT2 expression was significantly upregulated in the ELAVL1 antibody‐induced immunoprecipitation complex when compared with the IgG antibody‐induced complex (Figure 3B). The study also transfected ELAVL1 siRNA into AR42J cells to determine the consequent effects on SOAT2 expression. The data from Figure 3C indicated the high efficiency of ELAVL1 knockdown in AR42J cells. As shown in Figure 3D,E, ELAVL1 depletion downregulated SOAT2 expression at mRNA and protein levels in AR42J cells. Comparatively, the transcript half‐life of SOAT2 mRNA was shortened after ELAVL1 knockdown (Figure 3F). Further, we discovered that caerulein treatment upregulated ELAVL1 protein expression in a dose‐dependent manner (Figure 3G). The above data demonstrate that ELAVL1 stabilizes SOAT2 mRNA expression in AR42J cells.

### 3.4 SOAT2 overexpression attenuated ELAVL1 silencing‐induced effects in caerulein‐exposed AR42J cells

**FIGURE 4:**
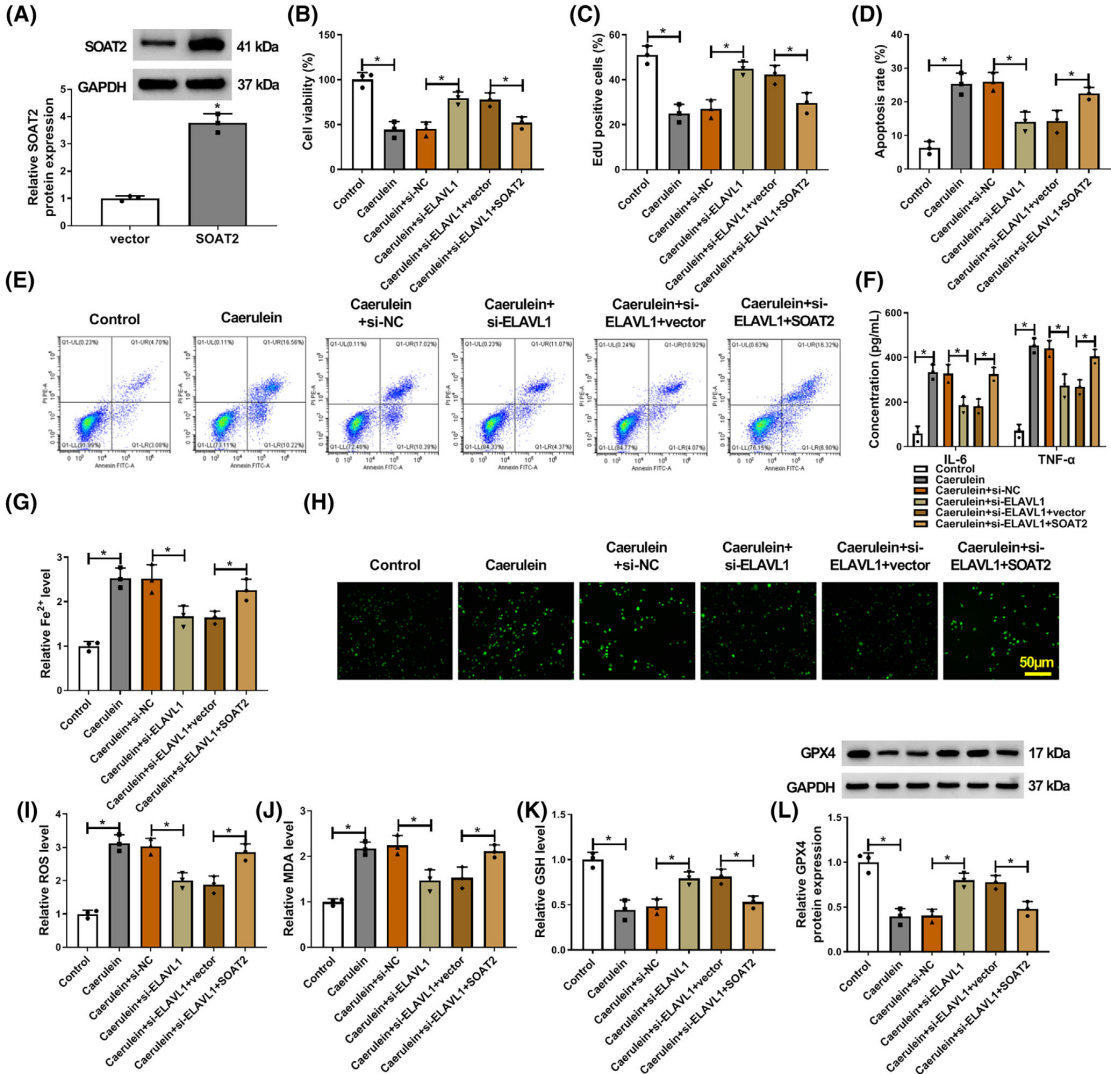
SOAT2 overexpression attenuated ELAVL1 silencing‐induced effects in caerulein‐exposed AR42J cells. (A) The efficiency of SOAT2 overexpression was analyzed by western blotting assay in AR42J cells. (B–L) AR42J cells were divided into control group, caerulein group, caerulein+si‐NC group, caerulein+si‐ELAVL1 group, caerulein+si‐ELAVL1 + vector group, and caerulein+si‐ELAVL1 + SOAT2 group. (B) Cell viability was assessed by the CCK‐8 assay. (C) Cell proliferation was analyzed by the EdU assay. (D and E) Cell apoptosis was assessed by flow cytometry. (F) ELISAs were performed to detect IL‐6 and TNF‐α levels. (G) Fe^2+^ colorimetric assay kit was used for Fe^2+^ level analysis. (H and I) ROS level was detected using a Cellular ROS Assay kit. (J) MDA level was analyzed using a lipid peroxidation MDA assay kit. (K) Glutathione detection assay was performed to analyze GSH level. (L) GPX4 protein expression was detected by western blotting assay. *p < 0.05.

Based on the above data, the study also studied whether the ELAVL1/SOAT2 axis regulated caerulein‐induced AR42J cellular injury. To this end, the study transfected ELAVL1 siRNA and SOAT2 overexpression plasmid before stimulating AR42J cells using caerulein. The result showed the high efficiency of SOAT2 overexpression in AR42J cells (Figure 4A). Subsequently, the results showed that ELAVL1 depletion promoted cell viability and proliferation and inhibited cell apoptosis in caerulein‐induced AR42J cells, whereas these effects were rescued after SOAT2 overexpression (Figure 4B–E). Moreover, ELAVL1 deficiency reduced IL‐6, TNF‐α, Fe^2+^, ROS, and MDA levels and increased GSH and GPX4 levels in caerulein‐exposed AR42J cells, however, these effects were rescued when SOAT2 expression was upregulated (Figure 4F–L). Thus, these results demonstrate that ELAVL1 silencing protects against caerulein‐induced AR42J cellular injury by downregulating SOAT2 expression.

### 3.5 ELAVL1 knockdown activated the NRF2/HO‐1 pathway by downregulating SOAT2 expression in caerulein‐exposed AR42J cells

**FIGURE 5:**
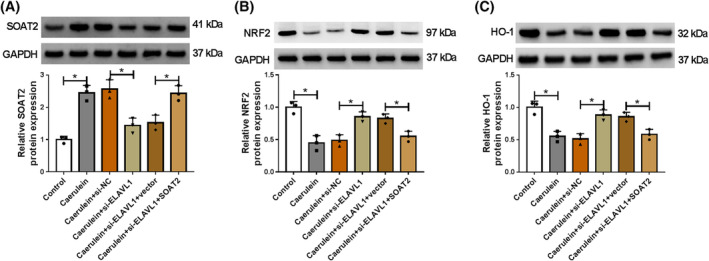
ELAVL1 knockdown activated the NRF2/HO‐1 pathway by downregulating SOAT2 expression in caerulein‐exposed AR42J cells. AR42J cells were divided into control group, caerulein group, caerulein+si‐NC group, caerulein+si‐ELAVL1 group, caerulein+si‐ELAVL1 + vector group, and caerulein+si‐ELAVL1 + SOAT2 group. (A–C) The protein expression of SOAT2, NRF2, and HO‐1 was analyzed by western blotting assay. *p < 0.05.

The study also analyzed whether ELAVL1 regulated the NRF2/HO‐1 pathway by upregulating SOAT2 expression in caerulein‐exposed AR42J cells. To achieve this, the study silenced ELAVL1 expression and overexpressed SOAT2 in AR42J cells, followed by stimulating the cells using caerulein. As shown in Figure 5A–C, ELAVL1 depletion inhibited SOAT2 protein expression and promoted NRF2 and HO‐1 protein expression in caerulein‐induced AR42J cells, whereas these effects were relieved after SOAT2 overexpression.

### 3.6 SOAT2 silencing protected AR42J cells from caerulein‐induced injury by increasing NRF2 expression

**FIGURE 6:**
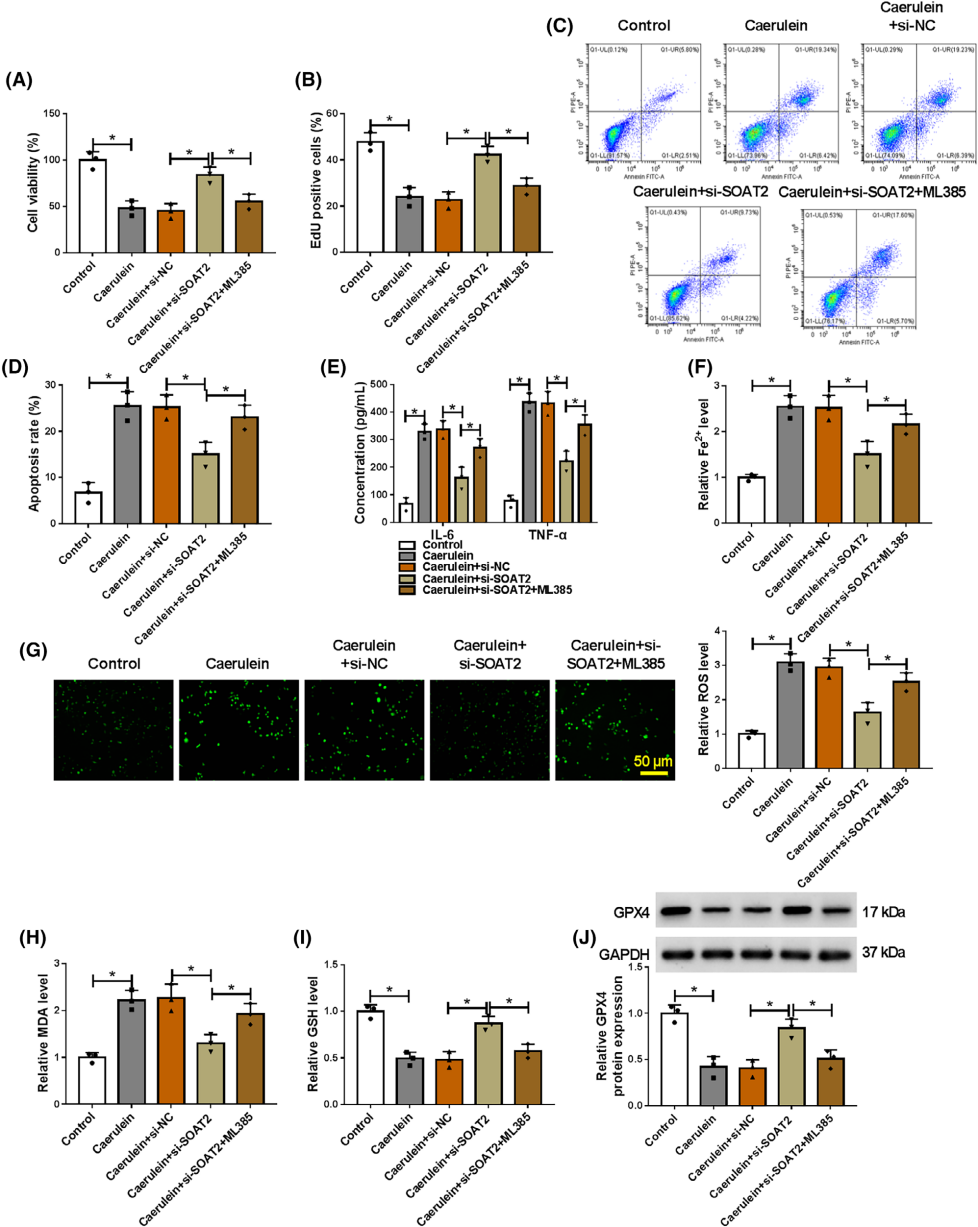
SOAT2 silencing protected AR42J cells from caerulein‐induced injury by upregulating NRF2 expression. AR42J cells were divided into five groups, including control, caerulein group, caerulein+si‐NC group, caerulein+si‐SOAT2 group, and caerulein+si‐SOAT2 + ML385 group. (A) Cell viability was assessed by the CCK‐8 assay. (B) Cell proliferation was analyzed by the EdU assay. (C and D) Cell apoptosis was assessed by flow cytometry. (E) ELISAs were performed to detect IL‐6 and TNF‐α levels. (F) Fe^2+^ colorimetric assay kit was used for Fe^2+^ level analysis. (G) ROS level was detected using a Cellular ROS Assay kit. (H) MDA level was analyzed using a lipid peroxidation MDA assay kit. (I) Glutathione detection assay was performed to analyze GSH level. (J) GPX4 protein expression was detected by western blotting assay. *p < 0.05.

The study further investigated whether the regulation of SOAT2 in caerulein‐induced AR42J cell injury involved NRF2. To achieve this, we silenced SOAT2 expression and then treated AR42J cells with NRF2 inhibitor ML385, followed by stimulation with caerulein. The results showed that SOAT2 depletion promoted cell viability and proliferation and inhibited cell apoptosis in caerulein‐induced AR42J cells, whereas these effects were rescued after ML385 treatment (Figure 6A–D). Moreover, SOAT2 deficiency reduced IL‐6, TNF‐α, Fe^2+^, ROS, and MDA levels and increased GSH and GPX4 levels in caerulein‐exposed AR42J cells, however, these effects were rescued when ML385 stimulation (Figure 6E–J). Thus, these results demonstrate that SOAT2 silencing protects against caerulein‐induced AR42J cellular injury by upregulating NRF2.

## 4 DISCUSSION

Acute pancreatitis is a severe inflammatory condition characterized by a complex molecular mechanism involving inflammatory responses, oxidative stress, iron death, and cell apoptosis.^27^, ^28^ AR42J cells are a type of rat pancreatic acinar cells commonly utilized in research to investigate acute pancreatitis.^29^ These cells provide a valuable tool for studying the disease due to their resemblance to human pancreatic cells and the ability to mimic certain aspects of acute pancreatitis in vitro. In this study, the focus is on analyzing the role and mechanism of SOAT2 in acute pancreatitis using AR42J cells. By investigating how SOAT2 impacts the pathogenesis of acute pancreatitis at the cellular level, researchers aim to elucidate its potential contribution to the disease pathology and identify new avenues for therapeutic intervention.

SOAT2 plays a critical role in lipid metabolism and is primarily involved in the acylation of cholesterol. A recent study has highlighted that SOAT2 expression is upregulated in a mouse model of acute pancreatitis.^11^ Herein, the authors also indicated its inhibitor reduced the levels of serum amylase, ameliorated pancreatic tissue pathological damage and downregulated ferroptosis‐related indicators. These data demonstrate that SOAT2 may contribute to the progression of acute pancreatitis. We analyzed its role in acute pancreatitis using caerulein‐induced AR42J cells. Our results showed its upregulation in caerulein‐induced AR42J cells. Consistent with the reported data,^11^ our results showed that downregulation of SOAT2 expression led to inhibition in ferroptosis‐related indicator (Fe^2+^) in the cell model. Ferroptosis can be caused by oxidative damage and involves the iron‐dependent accumulation of ROS and lipid peroxidation.^30^ We indeed discovered that SOAT2‐deficient AR42J cells displayed decreased levels of ROS and MDA and increased levels of GSH and GPX4. In addition, SOAT2 deficiency promoted proliferation and inhibited apoptosis and inflammatory response of caerulein‐induced AR42J cells. Thus, SOAT2 contributed to the progression of acute pancreatitis by inhibiting pancreatic exocrine cell proliferation and promoting apoptosis, inflammatory response, and ferroptosis.

RNA‐binding proteins (RBPs) constitute a diverse group of proteins characterized by their specific ability to interact with RNA molecules. This interaction plays a pivotal role in regulating a multitude of biological processes, notably including the stabilization of mRNA and the control of its translation into protein.^31^ ELAVL1 is a highly conserved family of RBPs that has been implicated in various diseases, including pancreatic disorders.^13^, ^32^ In particular, it has been reported that ELAVL1 interacts with AHR mRNA to increase gemcitabine sensitivity in pancreatic cancer cells.^33^ Moreover, ELAVL1 might stabilize mRNA of yes‐associated protein 1 to regulate pancreatic ductal adenocarcinoma cell migration.^34^ In terms of pancreatitis, we also knew that ELAVL1 was associated with pancreatitis‐like inflammatory microenvironment.^20^ Our data identified ELAVL1 as an RNA‐binding protein of SOAT2 and stabilized SOAT2 mRNA expression in AR42J cells. Caerulein‐induced AR42J cells showed an increase in ELAVL1 expression. ELAVL1 depletion attenuated caerulein‐induced inhibition in cell proliferation and promotion of cell apoptosis, inflammatory response, and ferroptosis. These findings suggest that ELAVL1 plays a promoting role in pancreatitis‐like cell injury. In particular, our data revealed that ELAVL1 regulated caerulein‐induced AR42J cell injury by upregulating SOAT2 expression.

Activation of the NRF2/HO‐1 pathway shows protective effects against inflammatory intestinal injury,^35^ cardiac injury,^36^ and pancreatic injury.^25^ However, whether the pathway participated in the regulation of the ELAVL1/SOAT2 pathway to caerulein‐induced AR42J cell injury has not been reported. Herein, we discovered that ELAVL1 depletion promoted NRF2 and HO‐1 protein expression in caerulein‐induced AR42J cells, whereas these effects were relieved after SOAT2 silencing. This result suggests that ELAVL1 inactivates the NRF2/HO‐1 pathway by regulating SOAT2 in caerulein‐induced AR42J cells. Subsequent results showed that NRF2 inhibitor relieved SOAT2 depletion‐induced effects in caerulein‐exposed AR42J cells. In terms of pancreatic injury, previous evidence has demonstrated that the NRF2/HO‐1 pathway ameliorates pancreatitis by regulating ferroptosis pathway, inflammation, and oxidative stress.^37^, ^38^ Thus, SOAT2 might regulate caerulein‐induced AR42J cell injury by inactivating the ELAVL1/SOAT2 pathway.

Taken together, ELAVL1‐dependent SOAT2 aggravated pancreatic exocrine cell injury by inactivating the NRF2/HO‐1 pathway in acute pancreatitis. However, the research scope is limited to the interaction between SOAT2, ELAVL1, NRF2, and HO‐1 in a cellular model (AR42J cells). The findings may not directly translate to clinical settings or other cell types, which can affect the generalizability of the results. In addition, the study relies heavily on an in vitro model using AR42J cells. The results obtained from such models may not fully reflect the complexity of the disease process in vivo, including the interactions between cells, the extracellular matrix, and the inflammatory milieu. Despite these limitations, the identified mechanism could complement current treatment approaches for acute pancreatitis, such as pain management, supportive care, and prevention of complications. By combining traditional treatments with targeted interventions that activate the NRF2/HO‐1 pathway, a more comprehensive approach to patient care might be achieved.

## CONFLICT OF INTEREST STATEMENT

The authors declare that they have no conflicts of interest.
